# Influence of the progression of pleural neoplasia on the outcome of pleurodesis in mice

**DOI:** 10.18632/oncotarget.27610

**Published:** 2020-05-26

**Authors:** Rodrigo Olivio Sabbion, Ricardo Mingarini Terra, Lisete Ribeiro Teixeira, Milena Marques Pagliarelli Acencio, Marcia Cristina Augusto, Priscila Berenice Costa, Paulo Manuel Pego Fernandes

**Affiliations:** ^1^Division of Thoracic Surgery, Instituto do Coracao, Hospital das Clinicas HCFMUSP, Faculdade de Medicina, Universidade de Sao Paulo, Sao Paulo, Brazil; ^2^Laboratorio de Pleura-Divisao de Pneumologia, Instituto do Coracao, Hospital das Clinicas HCFMUSP, Faculdade de Medicina, Universidade de Sao Paulo, Sao Paulo, Brazil

**Keywords:** pleural effusion, lung neoplasia, pleurodesis, metastasis, talc

## Abstract

Purpose: Experimental study aimed at evaluating whether pleural neoplastic disease is associated with the degree of pleural fibrosis over time caused by talc pleurodesis. The study describes changes in levels of inflammatory mediators and determines whether the course of time involved in progression of neoplastic pleural disease is the factor that influences safety of talc pleurodesis usage in mice.

Materials and Methods: Animals were randomized into two groups: Cancer group (CG) that received intrapleural injection of Lewis cells or Saline group (SG) that received saline injection. After, the animals were subdivided into Early (pleurodesis 3 days after pleural injection) and Late (pleurodesis 7 days after pleural injection) groups. Half of the animals in each group were euthanized 24 hours after pleurodesis (to obtain the inflammatory data); the remaining animals were killed after 8 days (to obtain the scores of pleural fibrosis).

Results: CGs had lower fibrosis scores than SGs comparing early phases to late phases. Inflammation scores were lower in CGs, particularly in Late group. In SGs the inflammation was intense in 100% of the animals.

In Late CG group pleural adhesions had the lowest scores; we found intense fibrosis only in SGs. VEGF and LDH levels had increased in animals with cancer, particularly in Late group. Systemic distribution of talc occurred only in Late CG.

Conclusions: The time for pleural neoplasia to evolve is inversely proportional to the degree of pleural fibrosis. Earlier pleurodesis yielded the best results related to fibrosis, with less systemic inflammation and is safer in mice.

## INTRODUCTION

Neoplastic pleural effusion is frequent and associated with quality commitment and life expectancy of patients due to the spread of the disease [1].

Pleurodesis arises in this context with the purpose of preventing the accumulation of fluid in the thorax, minimizing dyspnea and thus improving quality of life. Knowledge of the process and its failure factors are important to maximize the outcome of the procedure and to help in appropriate decision making.

Several factors may interfere with pleurodesis results, the presence of mesothelial cells exposed to the talc is fundamental for better effectiveness in promoting pleural symphysis. Studies suggest that when the mesothelial cell population is reduced the response to the sclerosing agent is proportionally decreased, worsening the final result of this procedure [2, 3]. However, the degree of neoplastic pleural involvement would not be solely responsible for the success or failure of pleurodesis when using talc. We do not know what other biological mechanisms may be involved in the failure of pleurodesis, particularly those related to the activation of the intrapleural inflammatory cascade, reflected by variations in TGF-β, IL-6 and VEGF levels.

There are no studies that directly correlate the degree of pleural involvement in malignant disease with the final result of pleurodesis. Therefore, the primary objective of this study was to evaluate whether the time course of neoplastic pleural disease is associated with the score of pleural fibrosis in mice submitted to talc pleurodesis. The secondary objectives were to describe the changes in pleural fluid levels of inflammatory mediators activated by pleurodesis at different times during the evolution of pleural neoplastic disease, as well as analyze whether the time course of neoplastic pleural disease is the factor that influences the safety of talc pleurodesis in mice.

## RESULTS

All animals tolerated anesthesia and surgery well. Four animals died during anesthesia and four after pleurodesis, probably due to surgical procedures.

### Fibrotic period

**Figure 1:**
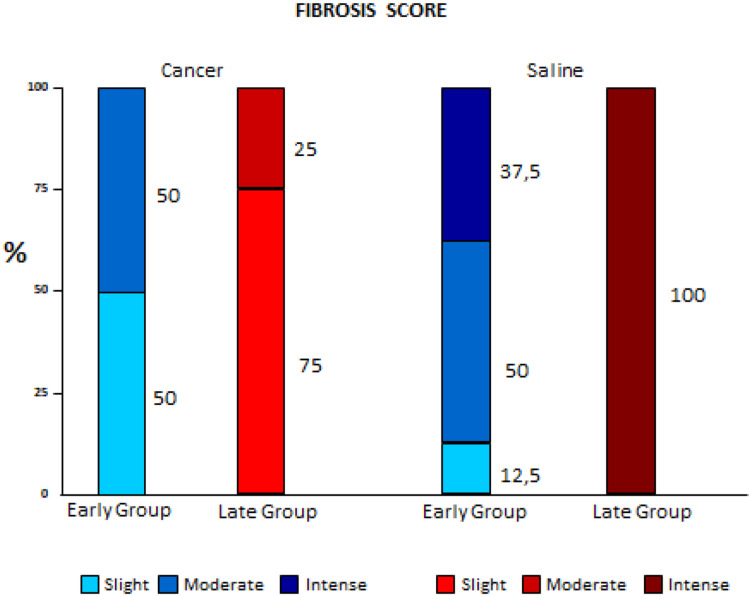
Comparison of fibrosis variation between early and late pleurodesis and between cancer and saline groups.

The Cancer groups had lower fibrosis scores than the Saline groups, and this difference was even more evident when comparing the early to the late phases (*p* < 0.001) (Figure 1).

**Figure 2:**
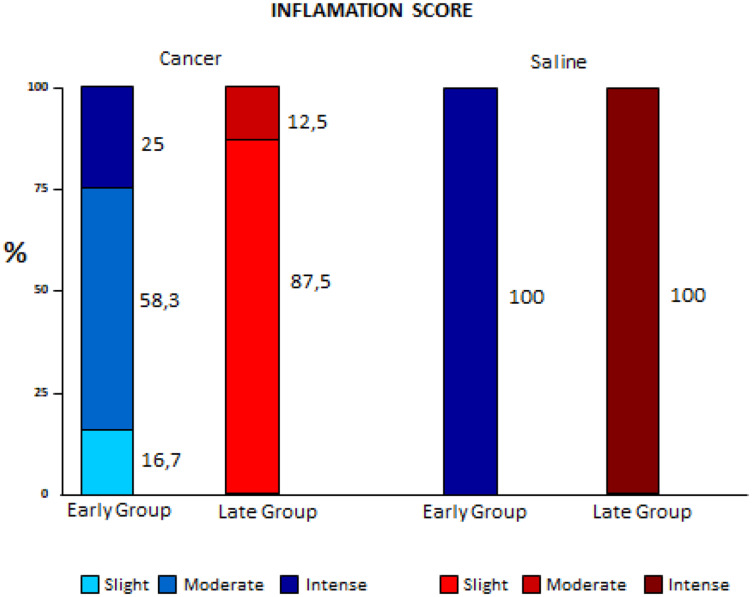
Comparison of inflammation variation between early and late pleurodesis and between cancer and saline groups.

With regard to inflammation scores, lower values were found in the Cancer group, which decreased even more in the Late Cancer group. In the Saline groups the inflammation was intense in 100% of the animals in both subgroups (early and late) (*p* < 0.001) (Figure 2).

**Figure 3:**
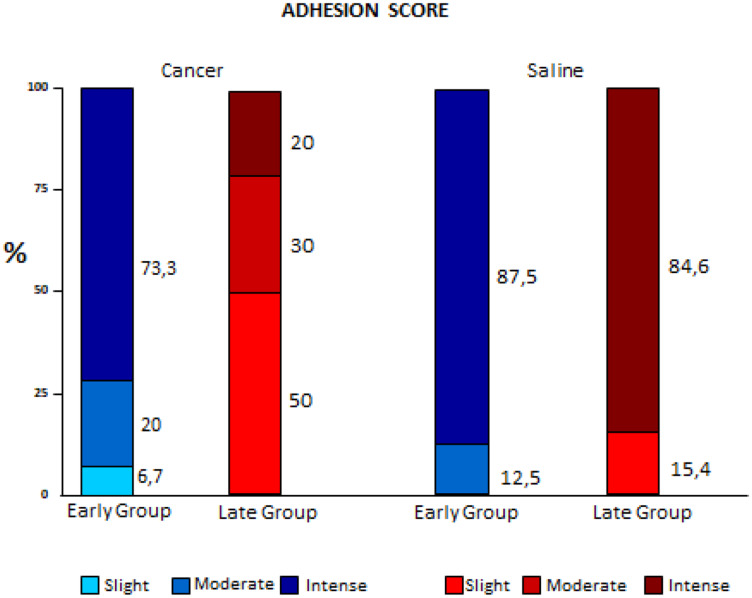
Comparison of variation of adhesion scores between early and late pleurodesis and between cancer and saline groups.

Adherences did not present statistical differences in the early stages. In the Late groups, the Cancer subgroup had the lowest scores; intense fibrosis was observed only in the Late Saline subgroups *(p* < 0.001). (Figure 3).

### Inflammatory period

**Figure 4:**
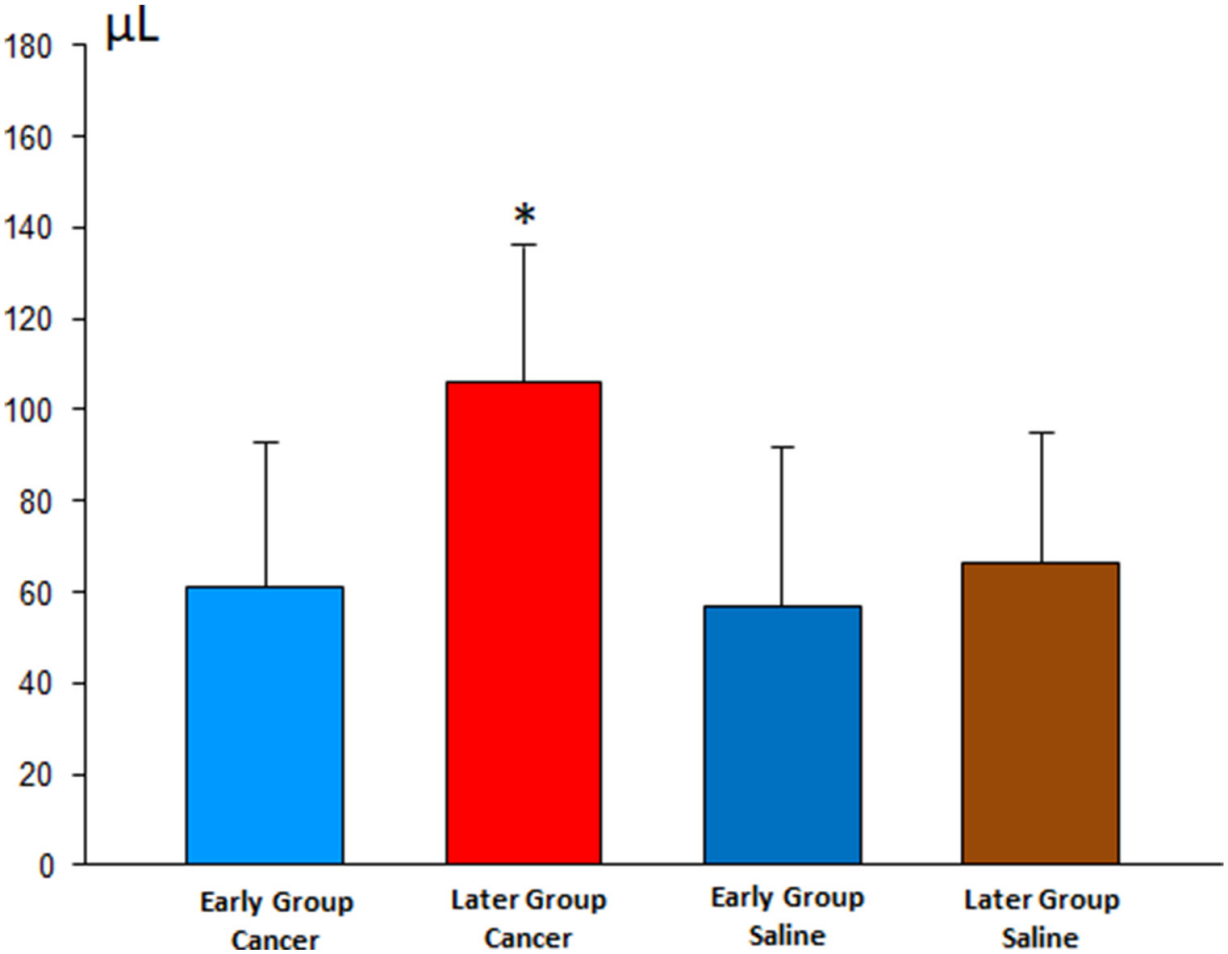
Volume of pleural effusion in the early and late stages of pleurodesis between the cancer and saline groups.

The analysis of the pleural inflammatory markers showed results with high variability; it was not possible to identify significant differences, except for VEGF, which increased in animals with cancer (particularly in the Late subgroup). This fact denotes a higher involvement of pleural disease, corroborating the finding of higher pleural fluid of the same subgroup, as shown in Figure 4.

### Systemic response

The systemic cellular response, as well as the systemic IL-6 in the animals of the Cancer and Saline groups did not demonstrate statistically significant differences. In contrast, systemic LDH was higher in the Cancer group with the highest indices in the Late subgroup. These results follow the numbers according to the increase of pleural disease in the Cancer subgroup.

Systemic distribution of talc occurred only in the late cancer group.

## DISCUSSION

This study showed that pleural fibrosis scores in the Cancer groups were significantly lower than observed in the Saline groups. This was more significant in late pleurodesis when compared to early pleurodesis, suggesting that neoplastic disease in the pleura would be associated with a lower occurrence of pleural fibrosis. Panadero et al. [4] suggested that the greater volume of tumor cells in the pleura would also imply a lower number of mesothelial cells exposed to the sclerosing agent, and therefore pleurodesis (mesothelial cell-mediated process) would also be impaired. Bielsa et al. [5], in a retrospective study of 450 cases of pleurodesis with talc, also obtained a negative correlation between malignant pleural disease and the end result of pleurodesis; however, according to logistic regressions, this isolated fact alone would not explain the failure of pleurodesis.

Sahn et al. [6] showed the lowest rates of fibrosis would be due to the low rates of glucose and pH [6, 7], mimicking in part, the neoplastic microenvironment; which could explain an interesting parallel finding in our study. A hundred percent of the Late Saline group presented severe fibrosis while only 37.5% of the Early Saline group had the same score. This could be explained by the fact that the time of pleural procedure in the Early group was shorter (only 72 hours), creating more inflammation and less fibrosis in the pleural microenvironment.

In a study with healthy pleura, after the instillation of talc, a rapid influx of neutrophils occurs in the pleural space in 24 hours [8]. However, there are no related studies of the neoplastic pleura. In our study the cell subtype that shows statistical difference was precisely in neutrophils: the highest indices obtained were in the Late Cancer and Early Saline groups. Although with a similar absolute neutrophils number the responses were extremely different, resulting in a lower fibrosis score in the Late Cancer group. According to Jantz and Antony [9] this could occur because in addition to activating the inflammatory cascade less, the lower number of mesothelial cells exposed to talc slurry would cause greater intrapleural fibrinolysis in order to regulate the balance of intrapleural coagulation, one of the first steps for the success of pleurodesis.

Adhesion scoring (macroscopic fibrosis) did not show a statistically significant variation between the Early groups (Saline and Cancer), showing that the initial phase of pleurodesis was similar in the Cancer and Saline groups. In Late groups 84.6% of the animals in the Saline group had intense adherence, whereas none of the Cancer group presented this score, demonstrating worse indices in advanced diseases, as suggested by the literature [2, 4].

VEGF participates in the formation of pleural effusions by increasing vascular permeability and potentiating the passage of liquid and proteins through capillaries and venules [10]. However, this fact also increases the passage of small molecules from the pleural space into the blood according to Gary Lee et al. [11]. There are no studies correlating VEGF levels with the end result of pleurodesis or the safety of talc related to systemic migration of particles.

We selected 24 hours after pleurodesis as an investigation point due to the peak action of pleural inflammatory cytokines and cell recruitment [12], however, we did not obtain differences to IL-6 and LDH. This may be related to several factors such as the minimum volume of pleural fluid obtained, the reliability of values obtained in the species studied, and the firing times of these enzymes and markers (which vary greatly). The exception was VEGF, higher in mice of the Cancer groups and which increased proportionally to the neoplastic pleural involvement. The highest indices were observed in the Late Cancer group. Zebrowski et al., who described much higher levels of VEGF in neoplastic fluids (ascites and liquor) than the same fluids not affected by neoplasia [13]. Our results may imply that VEGF reflects the greater amount of tumor and consequently a worse prognosis.

Analyzing the pleural fluid volumes of all animals (24 hs and 8 days after pleurodesis), we observed larger volumes in the late pleurodesis in the Cancer Group compared to early pleurodesis. This finding was similar to Zebrowski’s study [14] in which higher indices of VEGF have the highest volumes of pleural fluid.

The systemic response to pleurodesis was similar in the mice submitted to pleurodesis in the Cancer and Saline groups, apart from the analysis of the blood count in relation to the segmented ones, which was higher in the mice of the group Late Cancer. Studies suggested that the increase of neutrophils in the systemic circulation after pleurodesis could be due to the passage of talc particles or the passage of the cells themselves from the pleural cavity into the blood, reflecting pleural inflammation [15–17]. After analyzing our data we believe that it is more related to the circulation of talc, since we obtained similar values of neutrophils in the pleural fluid in the Salina subgroups, which did not reflect this increase in the systemic response. Thus, the greater pleural burden probably induces a greater systemic response or allows a greater penetration of the talc into systemic circulation. This can cause a more exacerbated systemic inflammatory response reflected by the systemic LDH with its higher indices obtained in the Late Cancer group, corroborating Swan et al. regarding the reflex of disease involvement not specifically adenocarcinoma [18].

As in previous studies [19] we found talc particles in the liver, spleen and kidney cuts, but only from animals with more pleural burden (Late Cancer group). We believe that this may also reflect the greater pleural permeability induced by VEGF (besides the greater presence of neoplastic cells in the pleura), which we discussed previously [14].

Our study has some limitations, the main one being that the subjects of this study were mice, with communicating pleural cavities and thus bilateral impairment and bilateral pleurodesis. Another fact that stands out as a limitation in this experimental protocol is the time of assessment of pleurodesis. In previous studies in experimental models of normal pleura evaluating different agents [20–22], the 28-day time was determined to be the most ideal. In this study of neoplastic pleura using LLC cells, the animals have a maximum survival time of 25 days [23], for this reason we used earlier assessment for pleurodesis.

According to our findings, the greater neoplastic pleural involvement (besides resulting in less collagen formation and consequently adhesions) may result in a lower pleural barrier in relation to talc dispersion due to a lower amount of mesothelial cells and higher indices of pleural cytokines [11, 24]. Among these, the main one would be the VEGF which with the increase in pleural permeability, leaves the channels more open and prone to passage of talc into the blood stream, thus furthering inflammation in the mouse and consequently becoming less safe.

## MATERIALS AND METHODS

### Study design

This is an experimental study using mice. All work was carried out at the Pleura lab in the Pulmonology Department together with the Thoracic Surgery Division within the University of São Paulo School of Medicine and associated with the Heart Institute (InCor) and the Clinics Hospital on the Medical School’s campus in São Paulo, Brazil. The use of animals was approved by the Ethics Committee.

**Figure 5:**
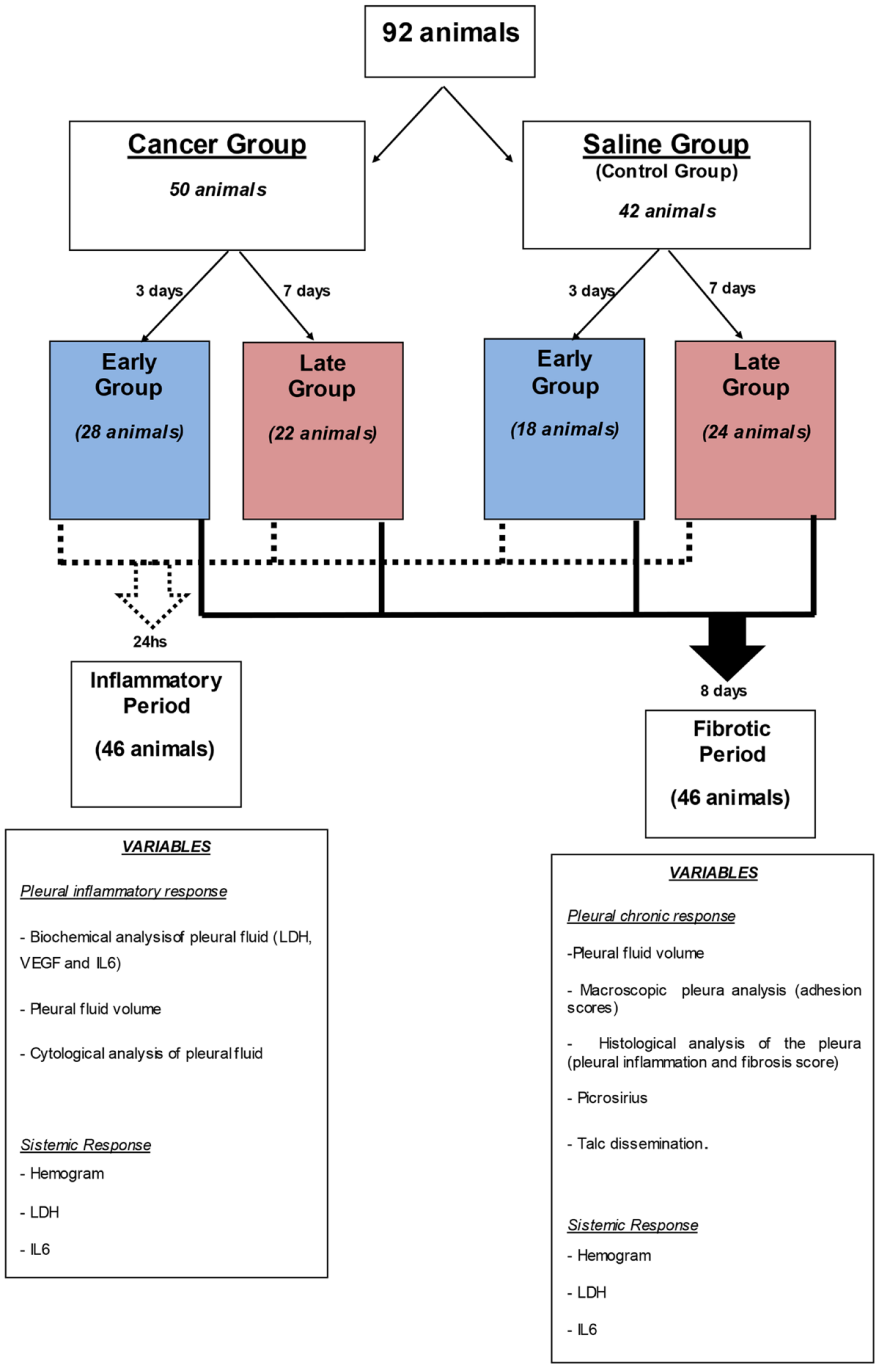
Algorithm of the subdivision of the groups, times and analyzes performed.

The choice of parameters to grade the outcome is expressed in the flow-chart Figure 5.

### Animal model

Ninety two 6-8 week-old male C57BL/6 mice were acclimatized for one week prior to beginning experimentation. The mice were divided into two groups: Cancer (n 50) and Saline (n 42) and given intrapleural injections of 50,000 Lewis Lung Carcinoma cells (LLC) or saline solution.

The Lewis Lung Carcinoma (LLC) cells were purchased from the American Type Culture Collection (Manassas, VA) and were cultured at 37°C in 5% CO_2_ –95% air using Dulbecco’s Modified Eagle Medium (DMEM) with 10% fetal bovine serum.

The Cancer and Saline groups were subdivided according to pleurodesis time after pleural injection (Figure 5). The Early Cancer and Saline subgroups were submitted to talc pleurodesis talc 3 days after intrapleural administration of Lewis cells or intrapleural saline solution. The Late Cancer and Saline subgroups were submitted to pleurodesis 7 days after intrapleural administration of LLC cells or saline solution. This was the time required to obtain a different degree of malignant pleural disease.

The pleural procedure on the animals was performed as described by Acencio et al. [23, 25].

Animals were anesthetized using 35 mg/kg of ketamine hydrochloride (Cristalia, Brazil) and 5 mg/kg of xylazine hydrochloride (Bayer, Brazil) prior to all procedures. The right chest was cleansed with an alcohol solution (Rioquimica, Sao Paulo, Brazil). The intrapleural injection was performed using a 23-gauge needle attached to a 1-mL syringe containing the solution of cells which was introduced into the chest cavity at 1 cm lateral to the right parasternal line. The plunger of the syringe was removed and the needle was slowly advanced until it reached the pleural space, where the sub-atmospheric intrapleural pressure allowed the fluid to enter the pleural cavity spontaneously. Subsequently, the system was closed so that inadvertent air intake did not occur, thus ensuring that pneumothorax did not occur during the procedure. The mice were monitored after the procedure until they were completely recovered.

After 3 days (Early groups) or 7 days (Late groups), the animals were again anesthetized and submitted to talc pleurodesis. A 21-G needle was inserted into the 5th right pleural space and 0.5 ml of sterile talc solution at a concentration of 400 mg/kg was injected. Upon injection conclusion the whole system was immediately removed to prevent inadvertent entry of air into the pleural cavity.

After pleurodesis, half of the animals in each group (Cancer and Saline) were euthanized in 2 different periods: The first period (inflammatory period) investigation to check for pleural inflammatory response and evaluate pleural fluid volume, pleural fluid cytology, biochemical and immunological analysis of the liquid (LDH, VEGF and IL-6), was performed 24 hours after pleurodesis. The second period (fibrotic period) investigation was carried out 8 days after pleurodesis, and evaluated pleural fluid volume, fibrosis scores, inflammation scores and pleural adhesion as well as the amount of collagen in the pleura (Picrosirius method). In both periods we evaluated the systemic response through complete blood counts, LDH and IL-6 and investigated the systemic spread of talc.

For euthanasia we used the same anesthetic solution, but with a 5-fold higher dose via intraperitoneal injection. After death the animal was positioned on the surgical table and a *xipho*-pubic *incision* was performed to gain full access to the organs.

The thorax was removed in block and the lungs expanded and fixed in 10% formaldehyde at room temperature.

The pleural fluid was collected by transdiaphragmatic puncture with a 19-G needle for volumetric quantification and analysis. Blood collection was performed by puncture of the inferior abdominal vena cava with a 19-G needle and filled into tubes with e*thylenediamine tetraacetic* acid (EDTA) anticoagulant, the same way as the pleural fluid was handled.

Liver, spleen and kidney were resected and transhilar cuts were made for tissue sampling. Subsequently the samples were fixed in 10% formaldehyde and held at room temperature for further analysis of talc spreading. The paraffin blocks were submitted to a 3 mm histological cut and stained with hematoxylin-eosin (HE) for analysis by optical microscopy and polarized light to investigate talc particles.

The fragments obtained obeyed standardization with the objective of homogenization of the samples. Fragments of the lower lobes were removed from the lungs. The other organs were sectioned transversely and fragments were removed, including the renal and splenic hila.

### Outcome evaluation

For evaluation and scoring of macro and microscopic fibrosis, the thorax removed in block was examined after 48 to 72 hours. This analysis was performed as described by Marchi et al. [26].

Macroscopic pleural adhesions were classified as scores 0–4, as follows: 0, absent; 1, less than three adhesions; 2, more than three localized adhesions; 3, dense adhesions diffusely distributed; 4, complete obliteration of the pleural space.

The microscopic score of fibrosis (0–4) was measured by the degree of the microscopic visceral pleural thickening in comparison to one control animal (normal monolayer of mesothelial cells) as follows: 0, absence of thickening; 1, thickening up to two times; 2, thickening from 2 to 5 times; 3, thickening from 5 to 10 times; 4, thickening greater than 10 times.

To assess the amount of collagen in the pleura of the animals, slides were stained with Sirius Red reagent (Picrosirius method), and we quantified collagen type I, type III and total using an image analyzer with specific software (Q500IW Imaging System Leica Ltd, Cambridge, UK), at the same location where we found most of the inflammatory elements.

The pleural fluid and blood were collected in tubes with the e*thylenediamine tetraacetic* acid anticoagulant (EDTA) for cytological evaluation (total cell count and leukocyte differential), IL-6 and VEGF (ELISA - *enzyme-linked immunosorbent assay*) and LDH (semi-automatic technique) according to the manufacturer recommendations.

Presence of talc particles in studied organs was investigated under 10× magnification using an image analysis program (LEYCA QWIN, USA); 10 random fields were evaluated. In each field the total area of the parenchyma and, through colorimetric detection, the area occupied by talc particles was quantified.

## CONCLUSIONS

The time it takes for pleural neoplasia to evolve is inversely proportional to the score of pleural fibrosis in mice submitted to pleurodesis with talc. The earlier the pleurodesis, the better the results related to fibrosis, less systemic inflammation, and lower circulation of talc; consequently, greater safety of the procedure.
